# Touch promotes emotional and autonomic recovery following virtual social exclusion in early adolescents

**DOI:** 10.3389/fpsyg.2026.1981624

**Published:** 2026-10-23

**Authors:** Letizia Della Longa, Irene Guglielminetti, Irene Valori, Francesco Rizzi, Gustavo Marfia, Teresa Farroni

**Affiliations:** 1 Developmental Psychology and Socialization Department (DPSS), University of Padova, Padova, Italy; 2 Centre for Tactile Internet with Human-in-the-Loop (CeTI), Chair of Acoustic and Haptic Engineering, Technische Universität, Dresden, Germany; 3 Department of Humanities, Social Sciences and Cultural Industries, University of Parma, Parma, Italy; 4 Department of the Arts, University of Bologna, Bologna, Italy; 5 Padova Neuroscience Center (PNC), University of Padova, Padova, Italy

**Keywords:** adolescent, auditory, cyberball, development, loneliness, social exclusion, touch, virtual reality

## Abstract

Social exclusion threatens the fundamental need for belonging and disrupts emotional and physiological regulation. This becomes particularly crucial during adolescence, when navigating complex social relationships is a key developmental challenge. Although gentle touch can reduce emotional distress and facilitate autonomic regulation, research on these effects in adolescents remains sparse. Here, we investigated whether tactile stimulation modulates adolescents’ responses to virtual social exclusion using an immersive virtual reality Cyberball paradigm combined with subjective and autonomic measures. Early adolescents (*N* = 67) experienced virtual ostracism followed by either affective touch or a control auditory stimulus. Compared with the auditory condition, affective touch was associated with a more positive post-exclusion affective state and lower perceived arousal. Although cardiac indices were not significantly altered by social exclusion itself, participants receiving touch showed lower heart rate, reduced sympathetic activation, and greater parasympathetic activity than those receiving auditory stimulation. Exploratory correlational analyses further indicated that these effects were more pronounced in adolescents reporting higher loneliness. Together, these findings suggest that touch supports emotional adjustment following virtual social exclusion and promotes a shift toward a more parasympathetic-dominant autonomic state during a developmental period characterized by heightened sensitivity to social evaluation.

## 1 Introduction

### 1.1 Social exclusion as a fundamental interpersonal stressor

Social exclusion refers to the experience of being ignored, rejected, or excluded by others, thereby disrupting opportunities to establish or maintain meaningful social relationships. According to the belongingness hypothesis (Baumeister and Leary, 1995), the motivation to establish and maintain stable, positive interpersonal relationships represents a basic psychological need that supports emotional well-being, physical health, and adaptive social functioning. Consequently, cues signaling the potential loss of social bonds are rapidly detected and prioritized, eliciting coordinated emotional, cognitive, behavioural, and physiological responses aimed at restoring social connection. Experimental research consistently demonstrates that even brief episodes of social exclusion undermine fundamental psychological needs, including belongingness, self-esteem, perceived control, and meaningful existence, while producing immediate emotional distress (Williams, 2009; Williams and Nida, 2011) Beyond these subjective experiences, exclusion heightens vigilance toward socially relevant cues and activates autonomic stress responses that facilitate monitoring of, and adaptation to, changes in the social environment (Hartgerink et al., 2015). Although these responses are generally adaptive in the short term, repeated or chronic experiences of exclusion can contribute to persistent feelings of loneliness and increase vulnerability to internalizing symptoms, including anxiety and depression (Hawkley and Cacioppo, 2010; Qualter et al., 2015). Importantly, the psychological consequences of social exclusion are shaped not only by the exclusion experience itself but also by the availability of mechanisms that promote recovery and restore emotional equilibrium (Williams and Nida, 2022). Identifying factors that facilitate adaptive regulation following exclusion is therefore essential, particularly during developmental periods characterized by heightened sensitivity to interpersonal experiences.

### 1.2 Early adolescence: a sensitive period for social exclusion

Early adolescence represents a particularly important developmental window for investigating the effects of social exclusion, as the need for peer acceptance becomes especially salient during this period. Early adolescence, which extends approximately from 10 to 14 years of age, represents a distinctive developmental stage that differs from both childhood and later adolescence in its neurobiological, physical, emotional, and social characteristics. The onset of puberty coincides with substantial brain reorganization, including widespread cortical thinning, reductions in gray matter volume, and ongoing maturation of white matter pathways that support increasingly efficient communication between brain regions (Tamnes et al., 2017; Parker et al., 2020; Fuhrmann et al., 2022). These neural changes occur alongside rapid bodily maturation and profound hormonal shifts, while socio-emotional systems involved in reward processing, social evaluation, and affective responding develop more rapidly than cognitive control systems (Steinberg, 2005; Casey et al., 2015). Consequently, early adolescents often exhibit heightened emotional reactivity and increased sensitivity to socially salient information compared with both children and older adolescents (Blakemore, 2008; Laursen and Veenstra, 2021). Alongside these neural changes, physiological systems involved in stress regulation also continue to mature during adolescence. In particular, developmental refinements in autonomic regulation support increasingly flexible physiological responses to emotionally salient social situations (Helánová et al., 2024; Šišáková et al., 2024). During socially evaluative contexts, adolescents show progressive specialization of sympathetic and parasympathetic responses, reflecting improvements in emotion regulation and adaptive social engagement (Hollenstein et al., 2012; Thayer et al., 2012; Beauchaine, 2015). Accordingly, social stressors such as peer rejection are consistently associated with reductions in heart rate variability, highlighting autonomic regulation as a sensitive marker of responses to social exclusion (Shahrestani et al., 2014).

A defining feature of this developmental period is the growing importance of peer relationships. As young adolescents gradually seek greater autonomy from their parents, peers become increasingly central sources of belonging, identity, and emotional support (Blakemore and Choudhury, 2006). At the same time, however, the need for peer acceptance renders young adolescents particularly sensitive to social evaluation and exclusion. Experimental and neuroimaging studies suggest that adolescence is characterized by heightened responsiveness to peer feedback and increased activation of neural systems involved in affective and social processing during experiences of social acceptance and rejection (Blakemore, 2008; Sebastian et al., 2010). Importantly, resistance to peer influence remains relatively limited during early adolescence and increases substantially only later in the second decade of life (Steinberg and Monahan, 2007), suggesting that the period between the age of 10 and 14 may be characterized by vulnerability to the effects of social stress.

This heightened social sensitivity may contribute to the emergence of loneliness, which has become increasingly prevalent among adolescents worldwide (Twenge et al., 2021). Loneliness is not merely the absence of social contact but rather the subjective perception that one’s social relationships are insufficient in quantity or quality (Hawkley and Cacioppo, 2010). Because peer belonging becomes a central developmental need during early adolescence, experiences of rejection, exclusion, or inadequate social support may be particularly impactful during this period (van Roekel et al., 2015). Longitudinal evidence further suggests that supportive friendships in early adolescence can promote resilient functioning and shape how social experiences are processed later in life, influencing both affective and neural responses to social inclusion and exclusion (Dauvermann et al., 2024). Repeated experiences of exclusion may therefore initiate a cascade of social and emotional difficulties, increasing vulnerability to persistent loneliness and subsequent mental health problems.

The ability to regulate the emotional consequences of social exclusion depends not only on cognitive processes but also on the integration of bodily signals that contribute to emotional experience. During adolescence, ongoing maturation of the brain systems supporting bodily awareness and self-regulation may influence how social experiences are processed (Klabunde et al., 2019; May and Tapert, 2025). Identifying bodily mechanisms capable of promoting recovery from social distress is therefore particularly relevant during this developmental period.

### 1.3 Affective touch as a regulator of social distress

Among the different forms of social interaction, affective touch occupies a unique position because it simultaneously conveys sensory information about the body and interpersonal information about the presence, intentions, and emotional state of another person (Burleson and Quigley, 2021). As such, affective touch is more than a tactile stimulus; it functions as a powerful social signal capable of communicating safety, affiliation, comfort, and support, particularly in situations of stress (Kidd et al., 2023). Across development, tactile interactions contribute to the differentiation and integration of bodily and social information, support interpersonal bonding, and scaffold the emergence of self-regulatory capacities (Fotopoulou and Tsakiris, 2017; Farroni et al., 2022; Suvilehto et al., 2023). Consequently, affective touch has been increasingly recognized as an important mechanism through which social exchanges regulate emotional and physiological responses to adversity.

The regulatory properties of touch arise from the interaction between its sensory and social components. At the sensory level, C-tactile (CT) afferents are a class of unmyelinated low-threshold mechanoreceptors predominantly distributed in hairy skin. They respond preferentially to gentle, dynamic stroking delivered at velocities of approximately 1–10 cm/s and at temperatures resembling those of human skin, characteristics commonly found in affiliative interpersonal touch (Ackerley et al., 2014; Löken et al., 2009; McGlone et al., 2014). According to the social touch hypothesis, the CT system is preferentially tuned to tactile signals that are likely to carry affiliative and socio-affective relevance, providing a peripheral pathway through which interpersonal touch may acquire emotional and motivational value (Morrison et al., 2010).

At the central level, CT-targeted stimulation is associated with activity in the posterior insular cortex. An activation-likelihood meta-analysis comparing affective and discriminative touch identified the posterior insula as the region showing the most selective and reproducible activation in response to tactile stimuli associated with positive hedonic value, supporting its central role in processing affectively relevant somatosensory information (Morrison, 2016). From this principal insular target, affective-touch processing extends to a broader distributed network that includes bilateral insular and parietal opercular regions, as well as somatosensory areas shared with discriminative-touch processing. Depending on the characteristics of the stimulus, the experimental task, and the interpersonal context, affective touch may additionally recruit regions involved in salience and interoceptive processing, reward, social perception, and mentalizing (Davidovic et al., 2019; Gordon et al., 2013; Kirsch et al., 2020; Sailer et al., 2016). More broadly, the involvement of neural systems supporting social perception and cognition is consistent with the social brain hypothesis, according to which the evolution and organization of the human brain have been shaped by the demands of navigating complex social relationships (Dunbar, 1998). Within this framework, CT-related input provides a bottom-up bodily signal that is integrated with top-down information concerning the identity and intentions of the toucher and the relational context in which touch occurs.

Affective touch has also been associated with autonomic and neuroendocrine regulation. Gentle stroking can promote vagally mediated parasympathetic activity, as reflected in reductions in heart rate and increases in heart rate variability, and physical contact has been linked to changes in stress- and affiliation-related hormones, including cortisol and oxytocin (Fairhurst et al., 2014; Triscoli et al., 2017; Holt-Lunstad et al., 2008; Pawling et al., 2017; Van Puyvelde et al., 2019).

Comparative evidence suggests that some of these regulatory functions are evolutionarily conserved: in non-human animals, affiliative tactile behaviours such as allogrooming strengthen social bonds and can buffer stress or provide comfort to distressed conspecifics (Kikusui et al., 2006; Wu et al., 2021). In humans, these conserved bottom-up sensory mechanisms are further integrated with higher-order information concerning the identity and intentions of the toucher and the relational context in which touch occurs. Although affective touch has frequently been conceptualized as an interoceptive modality because of its engagement of CT afferents and insular processing (Crucianelli and Ehrsson, 2023; Böhme, 2025) its affective and regulatory effects extend beyond the processing of internal bodily signals. Rather, they emerge from the integration of bottom-up tactile signals with higher-order social information. Accordingly, their magnitude critically depends on the interpersonal context in which touch occurs, including the identity of the interaction partner, the perceived intention of the toucher, relationship familiarity, and the social meaning attributed to the interaction (Ellingsen et al., 2016; Saarinen et al., 2021; Sailer and Leknes, 2022; Suvilehto et al., 2023).

Importantly, these sensory and social components are intrinsically intertwined during natural interpersonal interactions. Affective touch rarely constitutes a purely sensory event; instead, tactile stimulation is typically embedded within a relational context that conveys care, reassurance, affiliation, and emotional support. Consequently, the regulatory effects of touch are likely to emerge from the dynamic integration of bottom-up sensory signals with top-down cognitive and social interpretations, rather than from either component in isolation (Ellingsen et al., 2016; Sailer and Leknes, 2022).

Consistent with this integrative perspective, both the expectation and the experience of affective touch have been shown to attenuate emotional and physiological responses to stress (Kidd et al., 2023). Supportive physical contact from a close partner can reduce neural responses to threat and modulate physiological and neuroendocrine responses to acute stress, including blood pressure, cortisol, and alpha-amylase levels (Coan et al., 2006; Holt-Lunstad et al., 2008). Evidence from social-exclusion paradigms further indicates that slow, CT-optimal touch can mitigate exclusion-related threats to belonging, self-esteem, meaningful existence, and perceived control; importantly, broader changes in affect were also observed following non-CT-optimal touch, suggesting that the social presence of the toucher and the interpersonal context may themselves contribute to the soothing effects of tactile stimulation (von Mohr et al., 2017). Complementary naturalistic evidence emerged during the restrictions imposed in response to the COVID-19 pandemic, when deprivation of intimate touch was associated with greater anxiety and loneliness, as well as increased craving for interpersonal touch (von Mohr et al., 2021). Taken together, these experimental and observational findings support the role of interpersonal affective touch in facilitating emotional recovery and physiological regulation during stressful or socially adverse experiences (Farroni et al., 2022; Kidd et al., 2023).

While the regulatory effects of affective touch have been primarily investigated in adults, adolescence remains a relatively understudied developmental period. This gap is noteworthy because accumulating evidence suggests that adolescents may be particularly responsive to affective touch. Compared with children, they report greater pleasantness in response to CT-optimal stimulation, and affective touch appears to exert a stronger influence on socio-emotional processing during adolescence than in adulthood (Sehlstedt et al., 2016; Croy et al., 2019; Zingaretti et al., 2019; Della Longa and Farroni, 2025). Neuroimaging findings further indicate heightened recruitment of brain regions involved in reward, emotion, and bodily processing, suggesting that affective touch may be especially salient during this developmental stage (Bjornsdotter et al., 2014; May et al., 2014).

### 1.4 Rationale, aims, and hypotheses of the study

Although accumulating evidence indicates that touch facilitates recovery following social stress (Morrison, 2016), the mechanisms underlying this buffering effect remain poorly understood. The rationale for the present comparison arose from our previous study in adults, in which different forms of tactile stimulation—including skin-to-skin stroking, brushing, and tapping—facilitated affective and autonomic recovery following social exclusion despite differing in their sensory properties, CT-optimality, and degree of direct skin contact. By contrast, the no-touch condition was the only condition that consistently differed from the tactile conditions (Della Longa et al., 2026). These findings shifted the question from which specific tactile properties produce regulatory effects to what may make touch distinctive as a sensory experience: whether its effects arise because it directly engages bodily self-processing while simultaneously constituting interpersonal contact, or whether any salient sensory event may produce comparable recovery, for example by redirecting attention away from the preceding exclusion experience.

To address this question, we selected auditory stimulation as a salient but externally oriented sensory experience that did not involve direct stimulation of the participant’s body and did not convey the same inherent socio-affective meaning as interpersonal touch. Importantly, the tactile and auditory conditions differed across multiple dimensions, including sensory modality, bodily engagement, and social meaning. The present design was therefore not intended to isolate the specific contribution of each of these dimensions or to provide a perfectly matched sensory control. Rather, it compared two distinct categories of sensory experience to examine whether salient sensory stimulation per se would be sufficient to facilitate recovery, or whether the combination of bodily and interpersonal engagement characterizing touch would be associated with a different regulatory response. This question may be especially relevant during early adolescence, a developmental period characterized by heightened sensitivity to both social exclusion and interpersonal touch.

To address this question, the present study combined subjective measures of affective state (valence and arousal) with indices of cardiac activity, including heart rate (HR), heart rate variability (HRV), and markers of sympathetic and parasympathetic activation, to examine young adolescents’ responses to social exclusion induced using the VR Cyberball paradigm and their subsequent recovery during sensory stimulation.

Building on our previous work in adults (Della Longa et al., 2026), we compared affective touch with an auditory stimulus. While touch was directed toward the body and simultaneously conveyed bodily and interpersonal information, the non-social sound provided an externally oriented sensory event that lacked direct bodily contact and an inherently interpersonal component. The auditory condition was therefore included to examine whether a salient non-social stimulus could produce buffering effects similar to those previously observed following touch. It was not intended to isolate the contributions of touch’s sensory, bodily, and social properties.

Participants were not explicitly instructed to shift their attentional focus. Instead, sensory stimulation was delivered in a way that was expected to orient processing either toward the body and interpersonal contact through touch or toward the external environment through sound, while minimizing the use of explicit cognitive strategies. This comparison allowed us to assess the relative effectiveness of these two sensory experiences in promoting recovery following social exclusion, without attributing any observed difference to a single property distinguishing the conditions. Accordingly, we expected adolescents to show marked sensitivity to virtual social exclusion and predicted that tactile stimulation would facilitate affective and physiological recovery more effectively than auditory stimulation.

Furthermore, we included self-reported measures of loneliness to explore individual differences in responses to both social exclusion and sensory stimulation. We hypothesized that participants reporting higher levels of loneliness would show greater vulnerability to virtual social exclusion and derive greater buffering benefits from affective touch.

## 2 Method

### 2.1 Participants

Sixty-eight preteen participants (31 females and 37 males) took part in the experiment. All participants were between 9.9 and 13.9 years old (mean age 11.9 years, SD = 0.95). Figure 1 shows the distribution of participants’ age. Participants were recruited through invitations sent to families registered in the Babylab database at the University of Padova and through a middle school in Montegrotto (Padova). Parents provided written consent for their children to participate, after being informed about aims and procedures of the study. The participants were also informed about the study and provided written assent. The study was conducted in accordance with the ethical standards of the Declaration of Helsinki. The study protocol was approved by the Ethical Committee of Psychological Research at the University of Padova (code 5405).

**Figure 1:**
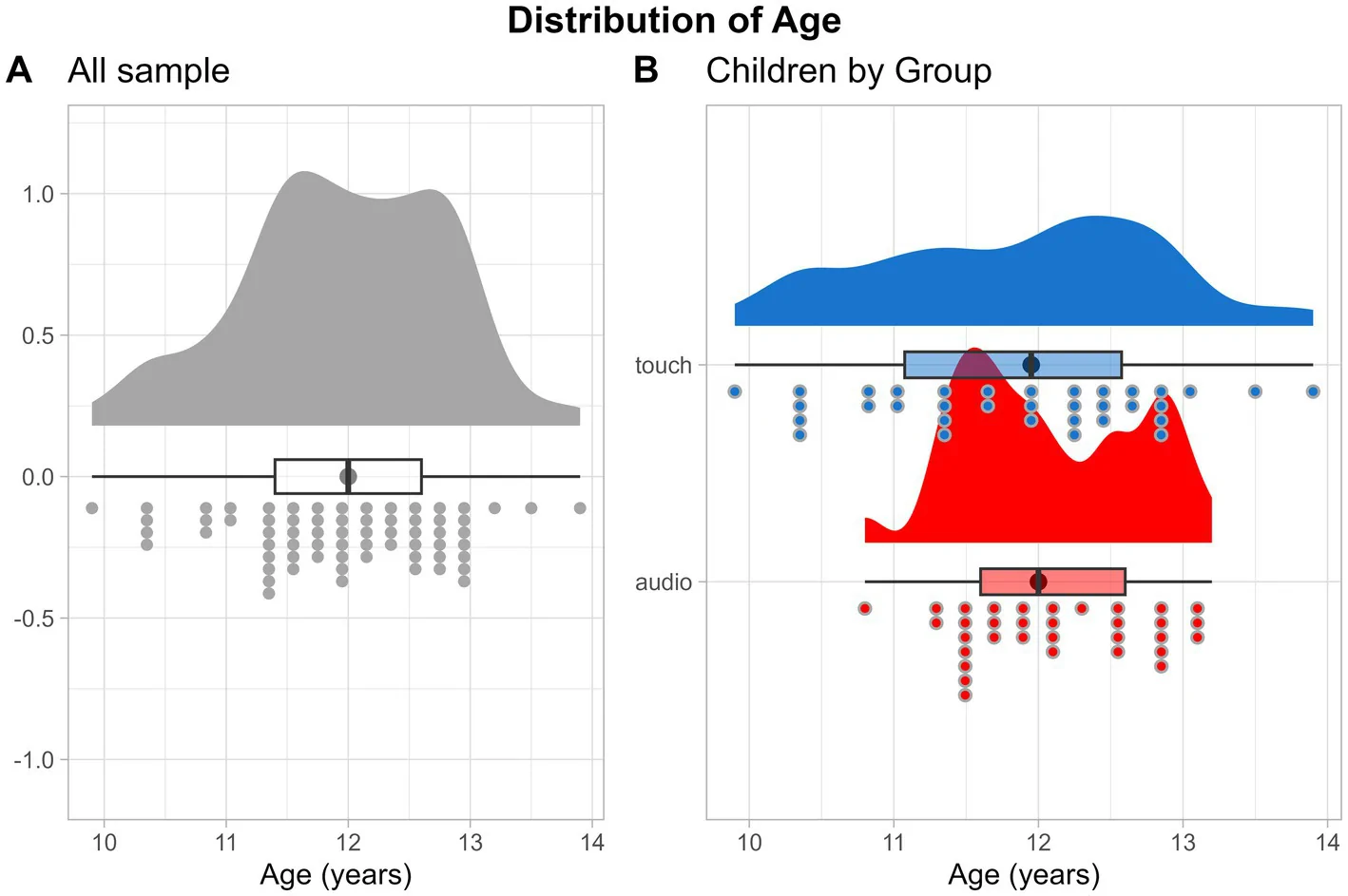
Age distribution of all participants **(A)** and participants divided in the two experimental groups **(B)**.

No formal *a priori* power analysis was conducted. Given the exploratory nature of the study and the absence of directly comparable evidence in young adolescents from which a reliable effect-size estimate could be derived, sample size was determined on the basis of recruitment feasibility, with the aim of including the largest possible number of eligible participants during the data-collection period. Previous studies conducted in adults using a related experimental design included 42 participants in each study and informed the general feasibility of the protocol, but were not used as the basis for a formal prospective sample-size calculation.

Although all participants completed every phase of the entire experimental session, the sample size for subjective and physiological analyses differed because of missing or unusable data. Specifically, for the subjective measures, participants responded to multiple-choice questions presented within the virtual environment by selecting their preferred option using the controller, which highlighted the corresponding response box. These responses were not automatically logged but were subsequently coded from recordings of the virtual sessions. Due to technical issues, the session from one participant could not be recorded. In addition, 13 participants (*N* = 6 in the touch group; *N* = 7 in the sound group) contributed only partially (8/10 items) to the subjective measures because some responses could not be reliably coded from the 2D video recordings. Specifically, movements of the participant (e.g., head or hand position) occasionally obscured the highlighted response option, preventing unambiguous identification of the selected answer. For the physiological measures, the data of 12 participants (*N* = 8 in the touch group; *N* = 4 in the sound group) were excluded because of artifacts in the cardiac recordings (e.g., movement-related noise). A detailed summary of data availability and exclusions for each measure is provided in Supplementary Table S1.

### 2.2 Stimuli and procedure

At the beginning of the experimental session, the experimenter placed three electrodes on the participant’s chest to record cardiac activity, ensuring their comfort throughout the procedure. The participant then donned a 360° VR headset (Meta Quest 2) while seated comfortably. During the preparation, an experimenter informed the participant that they would complete three activities in VR: a walk through a park, a ball-tossing game, and, depending on their assigned group, a session during which they would either receive tactile stimulation on the right forearm or hear auditory stimulation delivered through a speaker placed behind them. The total VR exposure lasted approximately 10 min. No participant spontaneously reported discomfort, nausea, dizziness, or other symptoms consistent with cybersickness, and no session was interrupted because of discomfort associated with the virtual environment. However, cybersickness was not assessed using a standardized questionnaire.

During the initial phase of the experiment, participants found themselves in an office-like virtual room, furnished with a desk and various objects (i.e., a plant, a magnetic board, a box and some cubes). They were instructed to familiarize themselves with the VR controllers by freely interacting with these objects (i.e., placing cubes in the box, moving magnets, watering the plant) thereby enhancing their sense of embodiment over the virtual hands, whose movements were congruent with those of the controllers. After completing this exploration, participants rated two statements regarding body ownership (“I feel that the virtual hands I see when looking down at myself are my own hands”) and agency (“I feel that the movements of the virtual hands are caused by my own movements”). Responses were recorded on a 6-point Likert scale ranging from −3 (strongly disagree) to + 3 (strongly agree). Subsequently, participants were teleported into a room where they completed five practice trials in the Cyberball paradigm with two computer-generated avatars (one male and one female). Specifically, a ball appeared in front of them along with instructions explaining that the ball would be thrown to either the left or the right avatar, depending on which hand they used to strike it.

Participants were then teleported into a virtual forest and instructed via a demonstration video to use the controllers to teleport along a path marked by waypoints. This neutral, motor-based task served to facilitate participants’ acclimation to the virtual setting and allow their cardiac activity to stabilize. The main experimental procedure, which was adapted from the Cyberball paradigm, then commenced. The experiment included three phases: inclusion, exclusion and sensory stimulation. Each phase lasted approximately 1 minute. Compared with our previous adult version (Della Longa et al., 2026), where inclusion and exclusion were presented in separate blocks, the adolescent adaptation implemented a single continuous game in which exclusion directly followed inclusion without interruption. This modification was designed to enhance the salience of the exclusion experience by fostering an initial expectation of social inclusion and group membership before its abrupt withdrawal.

Participants first encountered two computer-generated avatars (one female and one male) and were instructed to play a ball-tossing game with them. The game began with participants holding the ball and choosing to throw it to the left or right avatar by using the corresponding hand. The receiving avatar either returned the ball to the participant or passed it to the other avatar. During the first 30 tosses, all players had an equal likelihood of receiving the ball (inclusion phase). During the remaining 30 tosses the ball was passed exclusively between the two avatars, simulating an experience of social ostracism (exclusion phase). The fixed structure ensured that expectations of inclusion were first established before exclusion was induced. After the game, participants reported their subjective experience by answering two questions: “How often did you receive the ball?” (less, equal, or more than the others) and “How engaged did you feel in the game?” (1 = not at all, 5 = extremely).

Participants then remained in the virtual forest while receiving either tactile (touch group) or auditory (sound group) sensory stimulation. For participants assigned to the touch group, one trained female experimenter delivered tactile stimulation to the participants’ dorsal forearm over a 9-cm skin area in a proximal-to-distal direction. The stimulation consisted of gentle, hand-delivered stroking performed in a naturalistic manner with low pressure and at a velocity of approximately 3 cm/s, which falls within the 1–10 cm/s range associated with maximal CT-afferent firing and higher subjective pleasantness ratings (Löken et al., 2009; McGlone et al., 2014). Participants in the sound group heard an auditory stimulus consisting of an object-on-object stroking sound, obtained from a previous study (de Lagarde et al., 2025), made available on the Open Science Framework. The sound had been generated by stroking an aluminum tube against acrylic material at a speed of 6 cm/s. Using Audacity, we modify the stimulus by repeating a single stroking sound for 1 min, whereas the original version contained the sounds of three different stroking action (Experiment 3 - sound “stroking_object_3” https://osf.io/tx5ey/files/osfstorage?view_only=ce97e7ca72954eeb86ca89dd439dee56). The sound was presented through a speaker positioned 70 cm behind the participant. The volume was kept constant across all participants. Although the headset produced constant natural background sounds throughout the experiment (e.g., rustling leaves) at the same volume for all participants, the auditory stimulus remained clearly audible and distinguishable from these sounds.

In both sensory conditions, no visual information was associated with the tactile or auditory stimulation, and participants remained seated in the virtual forest.

Before and after the game, as well as after sensory stimulation, participants reported their affective state using the Self-Assessment Manikin (SAM), rating valence (1 = negative, 5 = positive) and arousal (1 = calm, 5 = highly aroused) (Bradley and Lang, 1994). Instructions and questions were presented within the virtual environment, and participants provided their responses by pressing the corresponding buttons with the controllers.

Physiological activity was continuously recorded throughout the experimental session. More specifically, cardiac activity during the inclusion phase was used as an individual baseline to assess subsequent physiological activation or deactivation associated with social exclusion and participants’ responses to the two types of sensory stimulation (Figure 2). We chose the inclusion phase as the reference baseline for three main reasons. First, our primary aim was to examine changes in cardiac activity elicited by social exclusion. Therefore, using the physiological state immediately preceding the exclusion phase allowed us to quantify individual physiological modulation relative to the most proximal pre-manipulation condition. Notably, previous meta-analysis showed that since early childhood, social engagement tasks, such as free play, teaching tasks and reunion with the caregiver, left HRV unaltered to a baseline. On the contrary disengagement tasks including the still face procedure and the separation phases of the strange situation, were associated with decreases in HRV (Shahrestani et al., 2014). Second, the inclusion, exclusion, and sensory stimulation phases all took place within the same virtual environment and task context, thereby providing a more controlled and comparable reference condition. In contrast, the initial forest exploration phase differed substantially in both task demands and contextual features. Third, the forest exploration was intentionally designed as a free-exploration period, resulting in considerable inter-individual variability in both duration and behavioral engagement. For example, some participants remained relatively stationary and explored the environment visually, whereas others moved more extensively and exhibited varying levels of familiarity with the teleportation controls. Consequently, physiological activity during this phase was likely influenced by heterogeneous exploratory behaviors, making it a less reliable baseline for subsequent comparisons. Finally, although heart rate can be influenced by motor activity, participants remained seated throughout the experiment and navigated the virtual environment exclusively using handheld controllers, thereby minimizing movement-related demands. Under these conditions, the inclusion phase provided the most appropriate and controlled reference point for evaluating cardiac changes associated with the social exclusion manipulation and the subsequent sensory stimulation phases.

**Figure 2:**
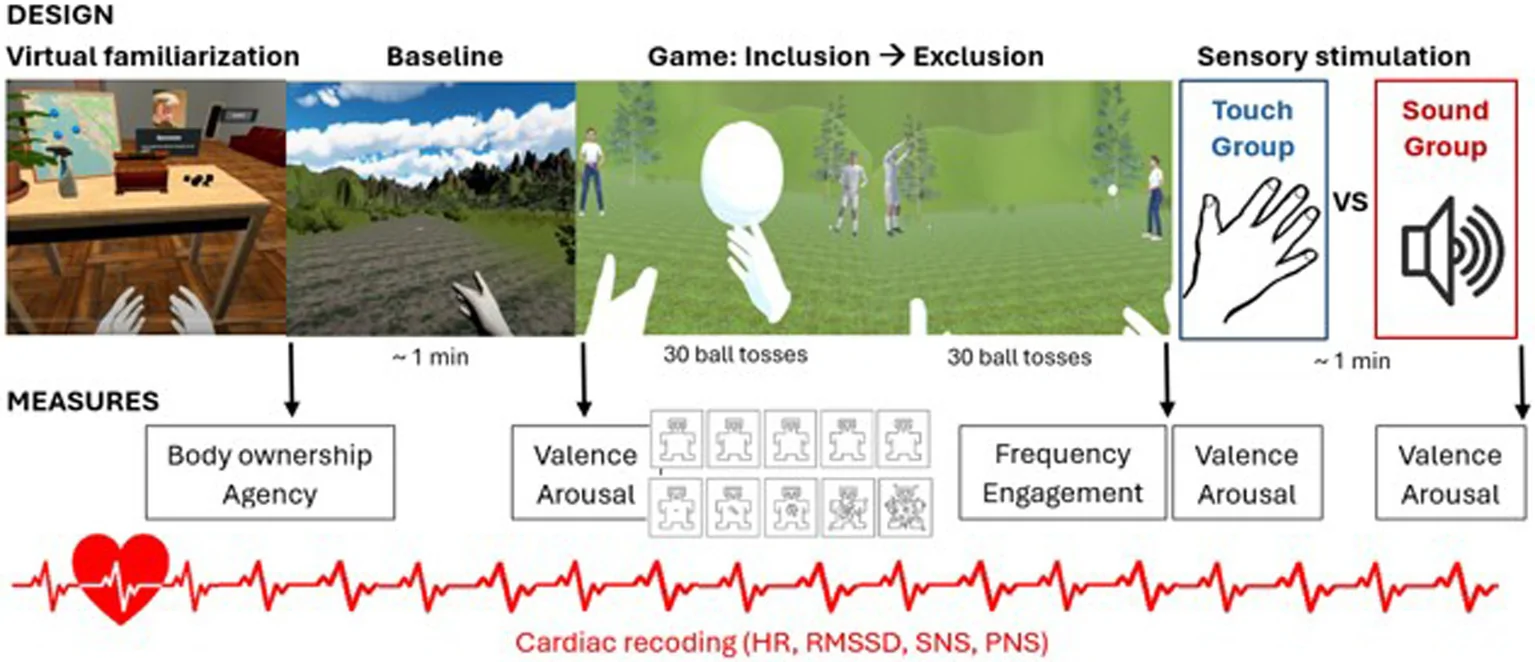
Schematic overview of the experimental procedure and timing of the measures. Following virtual-environment familiarization, participants rated body ownership and agency over their virtual hands. After an approximately 1-min baseline phase, they rated their valence and arousal and then completed the Cyberball game, comprising 30 inclusion and 30 exclusion ball tosses. After the game, participants reported perceived ball-toss frequency, engagement, valence, and arousal. They subsequently received 1 min of either affective-touch or auditory stimulation, according to group assignment, followed by a final assessment of valence and arousal. Cardiac activity was recorded continuously throughout the procedure and used to derive HR, RMSSD, and the Kubios-derived SNS and PNS indices.

At the end of the VR task, adolescents were asked to fill out the Children’s Loneliness Scale (CLS), a self-report questionnaire designed to assess feelings of loneliness among children, particularly in social and peer-related contexts (Asher et al., 1984). Because its items assess generalized perceptions of peer relationships rather than feelings experienced at a specific moment, the CLS is typically interpreted as a measure of relatively trait-like loneliness. However, a single administration does not provide information about the temporal persistence or chronicity of these feelings (Maes et al., 2017).

### 2.3 Electrophysiological data recording and processing

The electrocardiogram (ECG) was acquired using three Ag/AgCl electrodes positioned on each participant’s chests according to Einthoven’s Triangle, in a Lead II configuration (Anbalagan et al., 2023). Recordings were obtained using a multimodal physiological monitoring system that enables real-time biosignal acquisition (ProComp Infiniti; Thought Technology, Montreal, Canada), FDA-approved computerized recording device. The ECG signal was continuously sampled via a 12-bit analog-to-digital converter at 256 Hz and band-pass filtered between 0.3 and 150 Hz. ECG data were subsequently imported into Kubios HRV Scientific 4.1.0 (Oy, Kuopio, Finland; Tarvainen et al., 2014), where the interbeat interval (IBI) series were automatically extracted using the software’s built-in QRS detection algorithm based on the Pan-Tompkins method (Pan and Tompkins, 1985). Artefacts in the IBI series were corrected using automated preprocessing procedures followed by interactive visual inspection.

No direct recordings of sympathetic or parasympathetic neural activity were obtained in the present study. Instead, four ECG-derived measures of cardiac activity and autonomic modulation were examined: heart rate (HR), the root mean square of successive differences between adjacent heart periods (RMSSD), and the sympathetic nervous system (SNS) and parasympathetic nervous system (PNS) indices computed by Kubios HRV. HR is derived from the inter-beat interval (IBI) time series, expressed in beats per minute (bpm), and reflects the combined influence of parasympathetic (vagal) and sympathetic activity rather than providing a selective measure of either autonomic branch (Draghici and Taylor, 2016). Variations in HR may also be associated with motor and metabolic demands or with cognitive and attentional processes (Stern et al., 2000). Among heart rate variability (HRV) measures, RMSSD captures short-term beat-to-beat variability and is widely regarded as an index of pronominally vagally mediated cardiac modulation (Draghici and Taylor, 2016). The Kubios-derived PNS and SNS indices were included as complementary composite estimates of cardiac autonomic modulation (Tarvainen et al., 2014). The SNS index combines mean HR, Baevsky’s Stress Index, and the Poincaré-plot index SD2, which reflects longer-term variability in the RR-interval series. The PNS index combines mean RR interval, RMSSD, and the Poincaré-plot index SD1, which reflects short-term beat-to-beat variability. Higher PNS-index values indicate a cardiac pattern consistent with relatively greater parasympathetic modulation, whereas higher SNS-index values indicate a pattern consistent with relatively greater sympathetic modulation. Because these indices are derived from ECG and combine parameters that are not exclusively controlled by a single autonomic branch, they were interpreted as composite estimates rather than direct or selective measures of sympathetic and parasympathetic neural activity.

### 2.4 Statistical analysis

All statistical analyses were conducted using R (R Core Team, 2023). To evaluate the effects of the experimental manipulations on participants’ subjective affective states, we first analyzed self-reported valence and arousal on change scores from baseline. We then examined participants’ cardiac activity by assessing HR, RMSSD, SNS and PNS across the different phases of the experiment, and comparing responses to tactile and auditory stimulation. To assess potential baseline differences between groups, preliminary analyses were conducted using the baseline values of subjective measures (valence and arousal) and cardiac indices (HR, RMSSD, SNS, PNS; see Supplementary Table S2). Similarly, potential between-group differences in loneliness scores were assessed using the CLS (see Supplementary Table S3). Individual change scores for HR, RMSSD, SNS and PNS were subsequently computed by subtracting values obtained during the inclusion phase of the game from those obtained during each subsequent phase (i.e., exclusion and sensory stimulation) for each participant.

A model comparison approach was adopted to evaluate the contribution of several predictors of each dependent variable. Model fit and the relative statistical support for each model were assessed using the Akaike Information Criterion (AIC; Akaike, 1998) and AIC weights (Wagenmakers and Farrell, 2004). Specifically, we compared six candidate mixed-effects models comparing different combinations of the fixed effects of experimental Phase (two-level factor: exclusion and sensory stimulation), Group (two-level factor: touch group vs. auditory group), Phase × Group interaction, Gender (two-level factor: females vs. males), Age (continuous variable; see Table 1). Detailed reports of estimates from model comparisons and model testing are reported in the Supplementary material.

**Table 1:** Description of tested models: the random effect of participants was included in all models to account for the repeated-measures design.

Model	Factors included
Model 0 (null model)	Only random effect of participants: specified the hypothesis of no difference due to the independent variables and only accounted for individual variability
Model 1	Fixed effect of Phase
Model 2	Additive Phase and Group effects
Model 3	Interaction effect between Phase and Group
Model 4	Interaction effect between Phase and Group with additive Gender
Model 5	Interaction effect between Phase and Group with additive Gender and Age

Among the candidate models, we selected the one with the lowest AIC value (Hooper et al., 2008) and quantified its relative support using AIC weights, interpreted as the conditional probability that the model is the most plausible given the data and the set of candidate models (Wagenmakers and Farrell, 2004). The effects included in the selected model were then tested using analysis of deviance [ANOVA, type III Wald chi square test, “car” package (Fox and Weisberg, 2018)]. Mixed-effect models were employed to account for the repeated-measures design of the experiment, with observations from the different phases nested within participants (Hooper et al., 2008). Subjective and physiological measures were analyzed using the “lmer” function from the “lme4” package (Bates et al., 2015) and *p*-values were obtained with the “lmerTest” package (Kuznetsova et al., 2017). To quantify the models’ explanatory power, both conditional *R*^2^ (variance explained by fixed and random effects) and marginal *R*^2^ (variance explained by fixed effects only) were computed (Nakagawa and Schielzeth, 2013). Higher *R*^2^ values indicated that the model accounted for a greater proportion of variance in the dependent variable.

To explore potential associations among subjective affective states, physiological responses and self-reported loneliness, explorative correlational analyses were conducted using the “rcorr” function from the “Hmisc” package, which computed Pearson’s correlation coefficients for all pairwise combinations of variables. Given the exploratory and hypothesis-generating nature of these analyses, no correction for multiple comparisons was applied.

Finally, to visualize changes in the physiological measures over time, we fitted generalized additive mixed-effect models (GAMMs) using the “bam” function from the “mgcv” package. The models included a smooth effect of Time by Group and a smooth random effect for Participants. The degree of smoothness of the model terms was estimated as part of fitting. Model predictions were extracted and visualized using the “ggplot” function. For each participant, differential HR, RMSSD, SNS and PNS scores were computed by subtracting the participant’s mean values during the inclusion phase from each time-series observation. Accordingly, positive values indicate increases relative to baseline, whereas negative values indicate decreases relative to baseline. This procedure centres each participant’s time-series data around their individual baseline, thereby accounting for interindividual baseline differences.

## 3 Results

### 3.1 Preliminary analysis

At the beginning of the virtual experience, participants completed an embodiment phase in which they manipulated and interacted with virtual objects using VR controllers and their corresponding virtual hand representations. They were then asked to rate their sense of ownership of the virtual hands and agency over their movements. On a scale ranging from −3 (strong disembodiment) to +3 (strong embodiment), participants reported positive mean scores for both ownership of the virtual hands (M = 1.75, SD = 1.38) and agency over their virtual movements (M = 2.18, SD = 1.08; see Figure 3).

**Figure 3:**
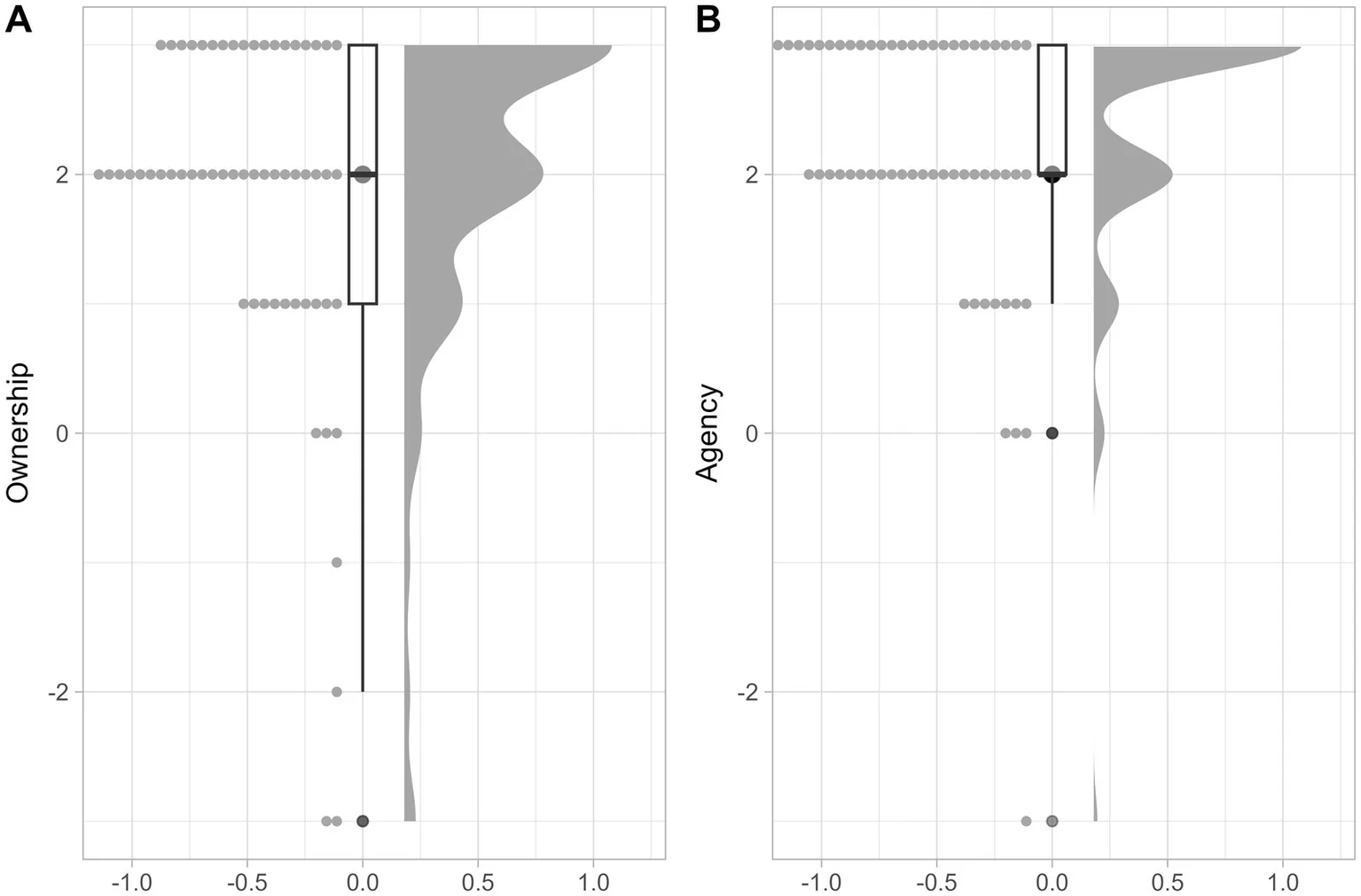
Distribution of scores for ownership over the virtual hands **(A)** and agency over the virtual movements **(B)**.

We also assessed whether participants experienced exclusion during the ball-tossing game. They rated their subjective perception of the number of ball tosses they received (more, equal, or fewer compared to the other players) as well as their perceived engagement in the game. Participants generally reported having received the ball less than the other players (M = −0.80, SD = 0.40), indicating that they were aware of being excluded from the interaction. They also reported moderate overall engagement in the game (M = 2.94, SD = 1.05; see Figure 4).

**Figure 4:**
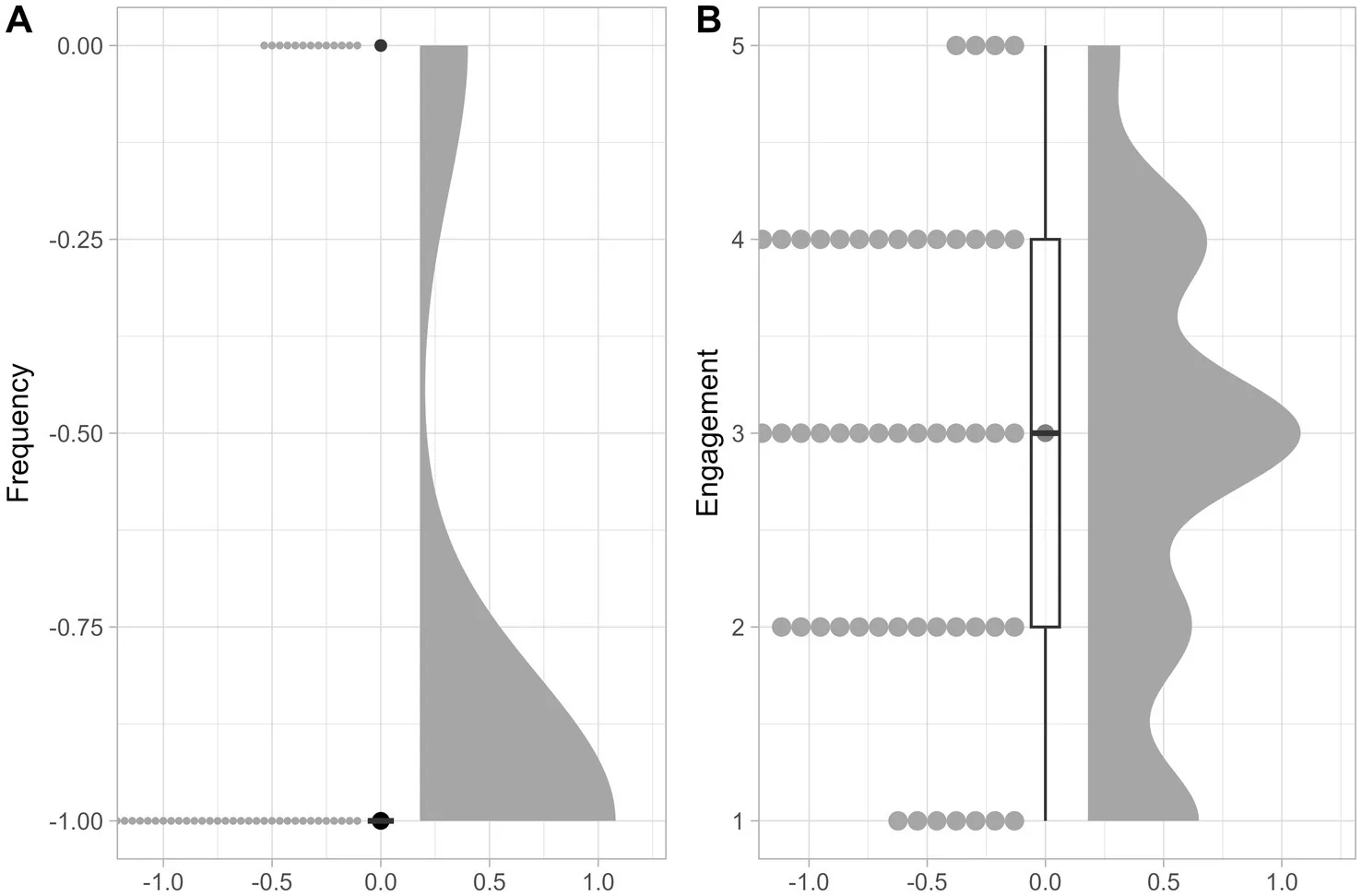
Distribution scores for frequency of perceived ball-tosses received **(A)** and engagement in the game **(B)**.

### 3.2 Self-report measures

To analyze the modulation of participants’ affective state in terms of valence (from negative to positive) and arousal (from very calm to very excited), we compared a set of six mixed-effect models as described in the Statistical Analyses section.

For the modulation of valence, according to AIC, AIC weights and likelihood ratio test, the best fitting model was Model 5 (AIC = 262.63, AIC weight = 0.533; *χ*^2^ = 3.285, *p* = 0.069, *R*^2^ conditional = 0.392, *R*^2^ marginal = 0.163; see Supplementary Table S4). The ANOVA on model 5 revealed a significant interaction effect between Phase and Group (*χ*^2^ = 5.87, *p* = 0.015). These results indicate that participants’ affective state decreased after experiencing exclusion from the game and was restored to baseline levels only for the group of participants experiencing tactile stimuli (Figure 5, panel A).

**Figure 5:**
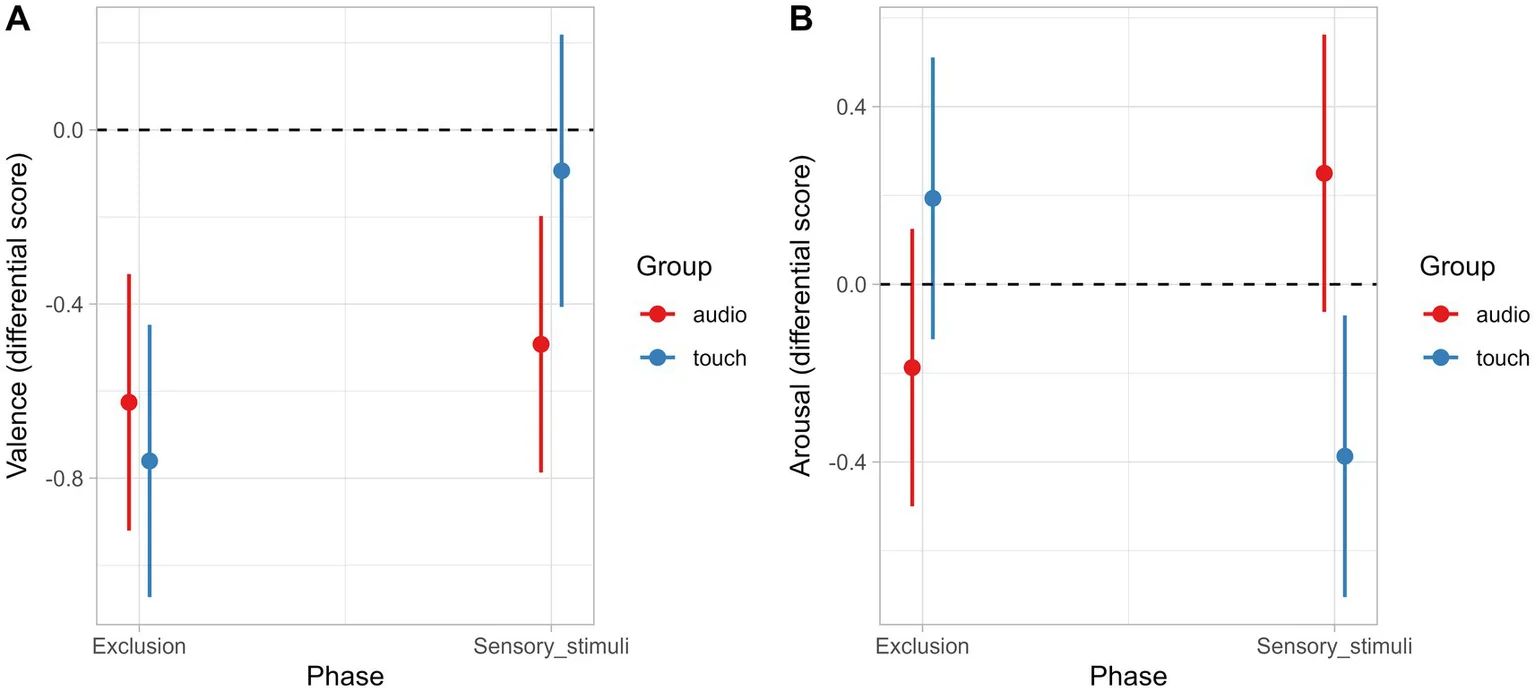
Plots of predicted values of valence **(A)** and arousal **(B)** modulation, showing the interaction effect between Phase and Group. Please note that we are considering differential scores compared to baseline levels (ratings before the game), thus positive scores indicate a relative increase in valence (more positive affective state) and arousal, while negative scores indicate a decrease in valence (more negative affective state) and arousal. Prediction lines and bars with confidence intervals (valence: Nobservations = 120; arousal: Nobservations = 126). Dashed lines indicate the pre-game baseline level of valence and arousal.

For the modulation of arousal, according to AIC, AIC weights and likelihood ratio test, the best fitting model was Model 3 (AIC = 315.74, AIC weight = 0.655; *χ*^2^ = 19.341, *p* < 0.001, *R*^2^ conditional = 0.570, *R*^2^ marginal = 0.081; see Supplementary Table S5). The ANOVA on model 3 revealed a significant interaction effect between Phase and Group (*χ*^2^ = 21.920, *p* < 0.001). These results indicate that subjective arousal decreased for participants experiencing the touch stimulation, while increased for those experiencing the sound stimulation (Figure 5, panel B).

To explore whether individual differences in loneliness were associated with responses to exclusion and sensory stimulation, we conducted exploratory correlational analyses between Children’s Loneliness Scale (CLS) scores and subjective measures of valence and arousal. No correction for multiple comparisons was applied; therefore, the reported coefficients and *p*-values are unadjusted and should be regarded as preliminary, hypothesis-generating findings requiring independent validation. Within the touch group, higher loneliness scores were associated with greater increase in valence (r = 0.23) and a greater reduction in arousal (*r* = −0.33) following tactile stimulation, see Table 2 and Figure 6.

**Table 2:** Correlation coefficients between Loneliness (CLS) and affective state (Pearson’s *r*).

	Game		Touch		Sound	
	Valence	Arousal	Valence	Arousal	Valence	Arousal
CLS	0.12	−0.14	0.23	−0.33	−0.17	−0.08

Correlations and associated *p*-values are uncorrected for multiple comparisons and should be interpreted as exploratory.

**Figure 6:**
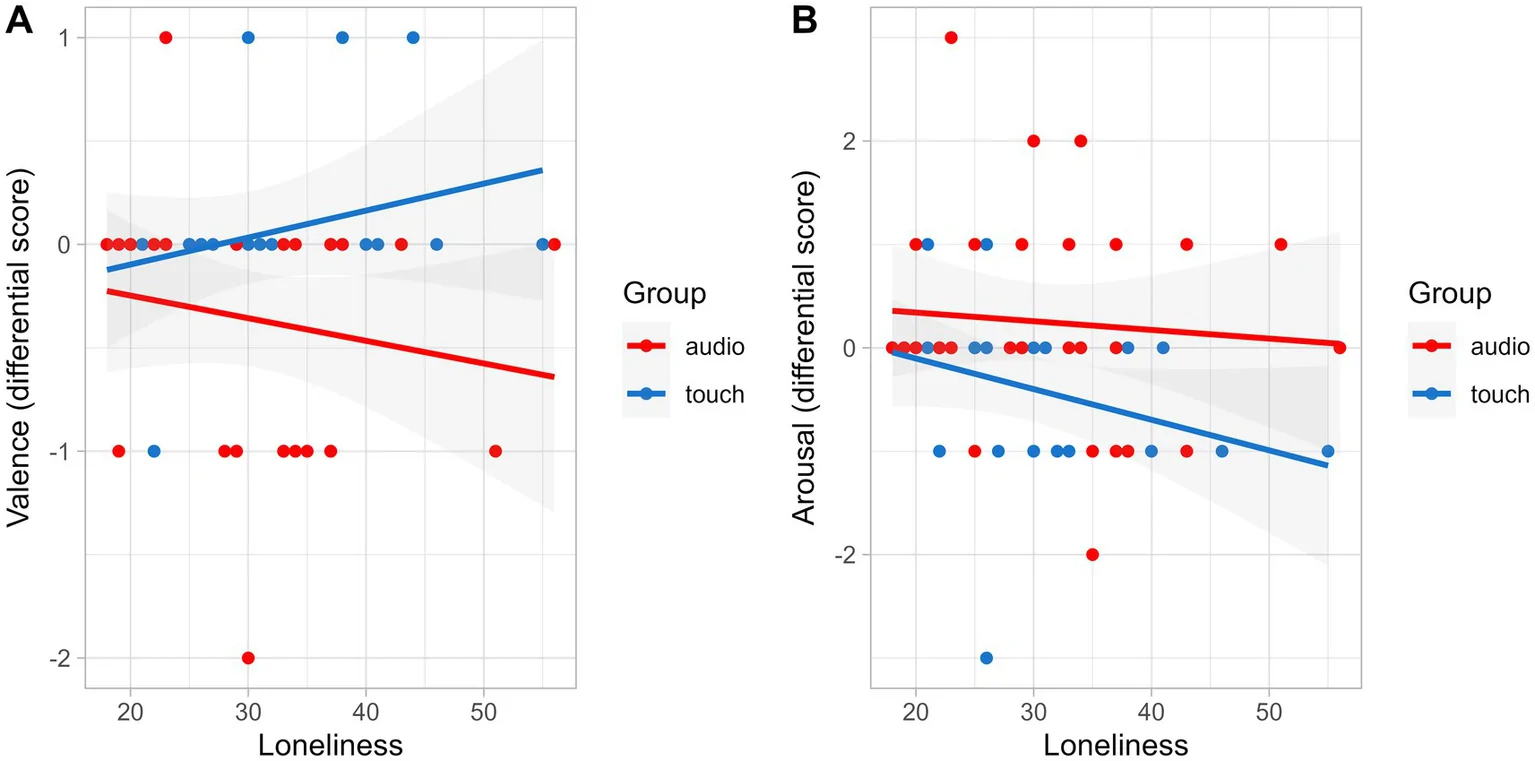
Plots showing Pearson’s correlation between Loneliness score (CLS) and subjective scores of valence **(A)** and arousal **(B)**.

### 3.3 Physiological measures

To analyze participants’ cardiac responses, we considered modulation of heart rate (HR), heart rate variability (RMSSD), sympathetic cardiac activity (SNS) and parasympathetic cardiac activity (PNS), and we compared a set of six mixed-effect models as described in the Statistical Analyses section.

For the modulation of HR, according to AIC, AIC weights and likelihood ratio test, the best fitting model was Model 3 (AIC = 591.26, AIC weight = 0.597; *χ*^2^ = 18.64, *p* < 0.001, *R*^2^ conditional = 0.687, *R*^2^ marginal = 0.196; see Supplementary Table S6). The ANOVA on model 3 revealed a significant interaction effect between Phase and Group (*χ*^2^ = 25.09, *p* < 0.001). These results suggest that participants experiencing touch stimulation showed a greater decrease in heart rate compared to those experiencing auditory stimulation (Figure 7, panel A).

**Figure 7:**
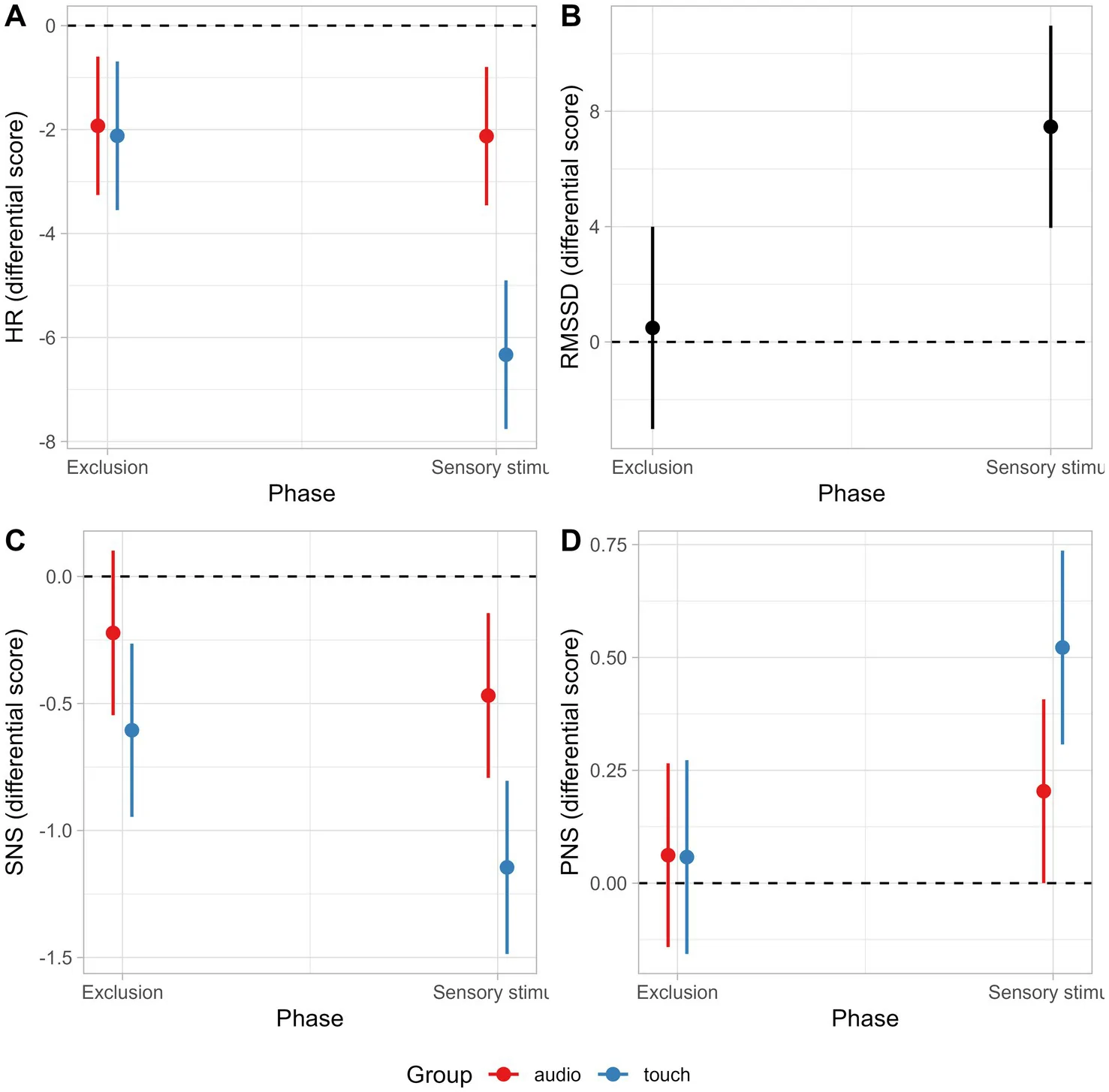
Plots of predicted cardiac modulations (heart rate and heart rate variability), showing the interaction effect between Phase and Group on HR **(A)**, SNS **(C)** and PNS **(D)** and the main effect of Phase on RMSSD **(B)**. Please note that we are considering differential scores compared to the inclusion phase. Prediction lines and bars with confidence intervals (HR N_observations_ = 112; RMSSD N_observations_ = 112; SNS N_observations_ = 112; PNS N_observations_ = 112). Dashed lines indicate the inclusion-phase reference levels of the cardiac measures.

For the modulation of RMSSD, according to AIC, AIC weights and likelihood ratio test, the best fitting model was Model 1 (AIC = 894.09, AIC weight = 0.390; *χ*^2^ = 11.37, *p* < 0.001, *R*^2^ conditional = 0.414, *R*^2^ marginal = 0.065; see Supplementary Table S7). The ANOVA on model 1 revealed a significant main effect of Phase (*χ*^2^ = 12.38, *p* < 0.001). These results indicate that participants showed an increase in HRV after being excluded, irrespective of the type of stimulation (Figure 7, panel B).

For the modulation of SNS, according to AIC, AIC weights and likelihood ratio test, the best fitting model was Model 4 (AIC = 203.47, AIC weight = 0.408; *χ*^2^ = 2.36, *p* < 0.125, *R*^2^ conditional = 0.804, *R*^2^ marginal = 0.205; see Supplementary Table S8). The ANOVA on model 4 revealed a significant interaction between Phase and Group (*χ*^2^ = 4.81, *p* = 0.028). These results suggest that participants experiencing touch stimulation showed a greater decrease of sympathetic cardiac activity compared to those experiencing auditory stimulation (Figure 7, panel C).

For the modulation of PNS, according to AIC, AIC weights and likelihood ratio test, the best fitting model was Model 4 (AIC = 142.96, AIC weight = 0.589; *χ*^2^ = 4.80, *p* = 0.028, *R*^2^ conditional = 0.482, *R*^2^ marginal = 0.182; see Supplementary Table S9). The ANOVA on model 4 revealed a significant main effect of Age (*χ*^2^ = 4.75, *p* = 0.029) suggesting that older adolescents presented higher modulation of parasympathetic activity. Moreover, an interaction between Phase and Group (*χ*^2^ = 5.488, *p* = 0.019) emerged. These results suggest that participants experiencing touch stimulation showed a greater increase in parasympathetic cardiac activity compared to those experiencing auditory stimulation (Figure 7, panel D).

To explore whether individual differences in loneliness were associated with cardiac responses to exclusion and sensory stimulation, we conducted explorative correlational analyses between Children’s Loneliness Scale (CLS) and changes in HR and RMSSD. As part of the same uncorrected exploratory analyses, associations were observed between loneliness and HR changes in both sensory-stimulation groups. Specifically, higher loneliness scores were associated with greater decrease in HR following tactile stimulation in the touch group (*r* = −0.31) and greater increase in HR following auditory stimulation in the sound group (*r* = 0.30), see Table 3 and Figure 8.

**Table 3:** Correlation coefficients between Loneliness (CLS) and affective state (Pearson’s *r*).

	Game		Touch		Sound	
	HR	RMSSD	HR	RMSSD	HR	RMSSD
CLS	0.04	−0.08	−0.31	−0.03	0.30	0.07

Correlations and associated *p*-values are uncorrected for multiple comparisons and should be interpreted as exploratory.

**Figure 8:**
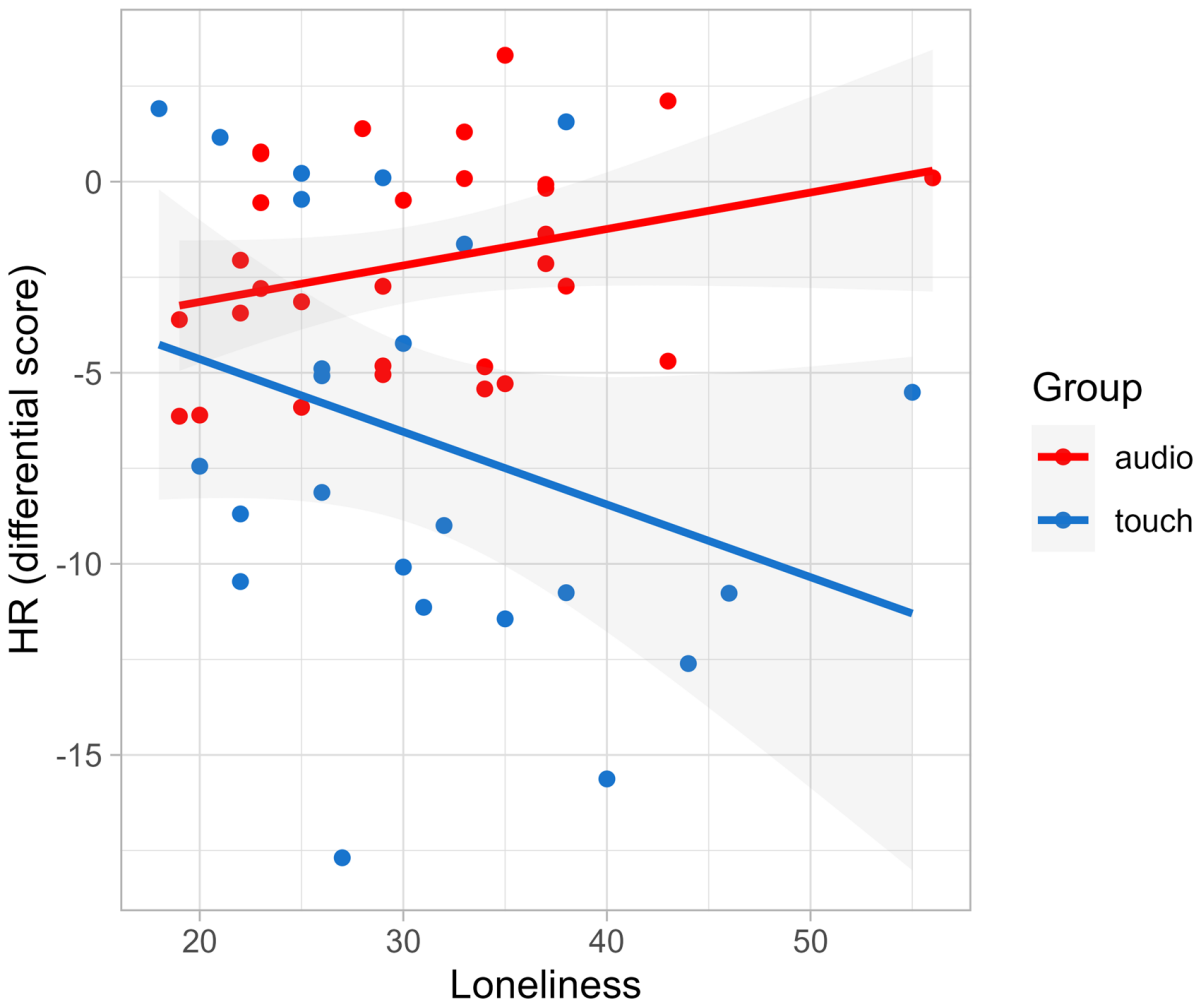
Plot showing Pearson’s correlation between Loneliness score (CLS) and modulation of HR during the sensory stimulation phase.

### 3.4 Time-series visualization

To explore changes in cardiac activity over time, we fitted generalized additive mixed-effects models (GAMMs) separately for HR, RMSSD, SNS, and PNS (Figure 9). Each model included group-specific smooth functions of time and a participant-level random-effect smooth. This approach allowed us to model potentially complex nonlinear temporal trajectories while accounting for repeated observations within participants. Moreover, the estimated smooth functions enabled us to visualize and compare changes in each cardiac measure over time across the two groups. The most pronounced divergence between the fitted trajectories emerged during the sensory-stimulation phase. Participants in the touch group showed a marked decrease in HR and in the Kubios-derived SNS index, accompanied by increases in RMSSD and the Kubios-derived PNS index. The auditory group showed changes of smaller magnitude, particularly for HR, SNS, and PNS. Toward the end of the stimulation phase, the trajectories tended to return toward the inclusion-phase reference level. These time-series models were used to describe and visualize the temporal evolution of cardiac responses.

**Figure 9:**
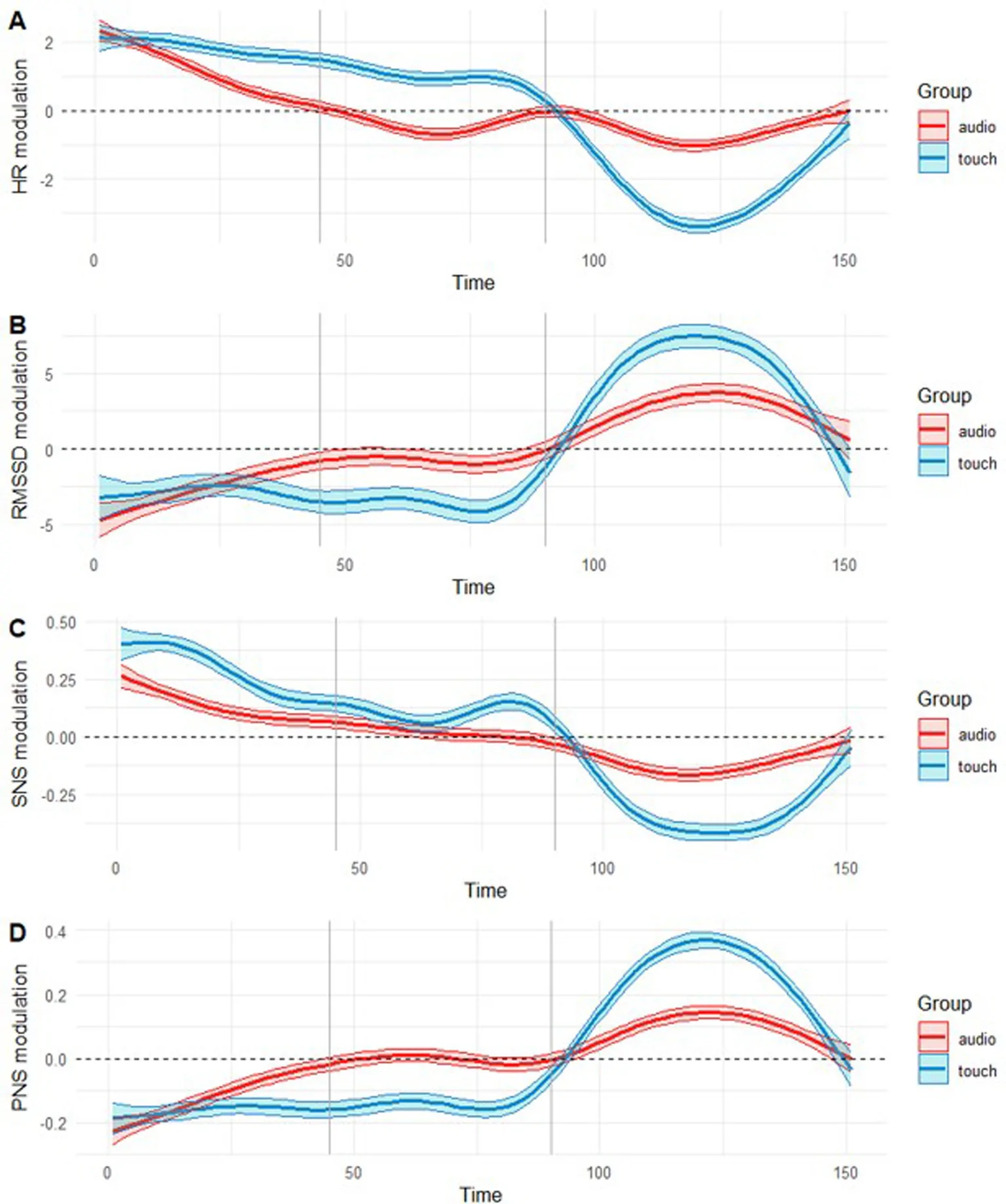
Group-specific temporal trajectories of HR **(A)**, RMSSD **(B)**, the Kubios-derived SNS index **(C)**, and the Kubios-derived PNS index **(D)**, estimated using generalized additive mixed-effects models. Solid lines represent the fitted smooth trajectories for the auditory-stimulation group (red) and touch-stimulation group (blue), and shaded bands represent the corresponding 95% confidence intervals. Vertical gray lines delimit the three experimental phases: inclusion, exclusion, and sensory stimulation. The horizontal dashed line indicates zero modulation relative to each participant’s mean level during the inclusion phase. Positive values indicate increases and negative values indicate decreases relative to this reference level.

## 4 Discussion

In the present study, we extended our previous findings in adults by investigating the role of touch in buffering the effects of virtual social exclusion in a sample of young adolescents, considering both subjective and physiological outcomes. Specifically, unlike our previous design, we compared affective touch—which combines socially relevant tactile cues with bodily and interoceptive processing—with an auditory stimulus that primarily engages exteroceptive processing. This comparison was intended to examine whether the regulatory effects of touch reflect a general consequence of exposure to a salient sensory stimulus or instead depend on properties that are unique to interpersonal affective touch.

Unlike non-social auditory stimulation, affective touch simultaneously engages the body as the target of sensory experience while conveying a social signal of proximity, care, and interpersonal connection. During natural interactions, these bodily and relational dimensions are intrinsically intertwined, making affective touch more than a simple sensory event. By contrast, the auditory control stimulus provided a salient sensory input without involving direct bodily contact nor communicating interpersonal closeness. This distinction allowed us to test whether a sensory stimulus that simultaneously engages the body and signals social connection possesses an intrinsic regulatory value beyond that attributable to sensory stimulation alone. Accordingly, we hypothesized that affective touch would facilitate recovery following social exclusion more effectively than auditory stimulation by promoting both bodily engagement and a renewed sense of social connectedness, thereby reducing subjective and physiological indices of social stress.

Adolescent participants demonstrated clear sensitivity to social exclusion within the virtual game. Despite the interaction occurring in a simulated environment, they detected the imbalance in social participation, consistently reporting that they had received fewer ball tosses than the other players. This subjective appraisal was paralleled by a decline in affective state: subjective valence ratings significantly decreased from pre- to post-exclusion, as indexed by negative change scores. Thus, even brief, digitally mediated exclusion elicited a measurable reduction in subjective well-being in this sample of young adolescents. Crucially, the subsequent sensory manipulation differentially shaped recovery. Following affective touch, subjective valence returned toward baseline; whereas participants exposed to auditory stimulation remained in a comparatively more negative affective state. The groups also diverged in arousal: touch was associated with decreased arousal—consistent with a potential calming effect—whereas auditory stimulation was accompanied by increased arousal. These findings suggest that the beneficial effects of touch cannot be readily attributed to exposure to a salient sensory stimulus per se. Rather, they support the view that tactile stimulation involving direct bodily contact and conveying interpersonal proximity may possess a distinctive capacity to facilitate emotional recovery following social exclusion. While the present design does not allow us to disentangle the relative contribution of bodily engagement and social meaning, it indicates that their combination confers a regulatory advantage beyond that provided by auditory sensory stimulation alone.

At the physiological level, cardiac indices did not reveal the expected autonomic response to social exclusion itself, as neither HR nor HRV showed the anticipated stress-related pattern during the exclusion phase. This finding suggests that the virtual manipulation was not sufficient to elicit a robust autonomic stress response, at least as indexed by the cardiac measures employed. Nevertheless, a consistent pattern emerged during the subsequent recovery phase. Heart rate and the composite sympathetic nervous system (SNS) index converged in showing that participants receiving affective touch exhibited a stronger autonomic downregulation than those exposed to auditory stimulation, characterized by lower heart rate and reduced sympathetic activation. Although RMSSD increased across the recovery phase regardless of sensory condition, suggesting a general recovery process, analyses of the composite parasympathetic nervous system (PNS) index revealed a significant Phase × Group interaction, indicating that parasympathetic recruitment was more pronounced following affective touch than auditory stimulation. Taken together, the convergence between heart rate, SNS, and PNS measures points to a coordinated shift toward a more parasympathetically dominated autonomic state after affective touch. Rather than demonstrating stronger physiological reactivity to social exclusion, the present findings indicate that affective touch facilitates autonomic recovery by simultaneously promoting parasympathetic engagement and sympathetic withdrawal.

Importantly, exploratory correlational analyses suggest that individual differences in loneliness may be associated with responses to sensory stimulation. Adolescents reporting higher loneliness showed more pronounced divergences between touch and sound at both subjective and physiological levels. Within the touch group, higher loneliness scores were positively associated with increases in valence responses and greater decreases in arousal and heart rate. Although these associations should be interpreted cautiously, this pattern may indicate that adolescents reporting higher loneliness experienced greater affective and autonomic changes following touch. One possible interpretation is that a sensory experience simultaneously involving direct bodily contact and conveying interpersonal closeness becomes particularly salient for individuals whose need for social connection is less fulfilled. Rather than simply providing a salient sensory input, affective touch may therefore acquire greater regulatory value when social connectedness is perceived as lacking. By contrast, in the auditory condition, loneliness was positively correlated with HR, suggesting that higher loneliness was associated with physiological recovery in response to a sensory stimulus lacking direct bodily and interpersonal components. This pattern is broadly consistent with previous evidence linking loneliness to heightened vigilance for social information (Cacioppo et al., 2016; Qualter et al., 2013), while extending these observations to autonomic responses elicited by different forms of sensory stimulation. Although these findings are exploratory, they raise the possibility that loneliness influences not only social-cognitive processing but also the regulatory value attributed to sensory experiences differing in their capacity to engage the body and convey social connection.

Because affective touch simultaneously engages the body and conveys interpersonal proximity, its regulatory effects may be especially relevant when the need for social connection has been threatened. Rather than acting solely as a salient sensory stimulus, touch may provide a bodily experience of social connectedness that promotes emotional and physiological readjustment. This interpretation is consistent with evidence in adults showing that physical contact reduces perceptions of relational neglect, particularly among individuals with limited opportunities for intimate touch (Heatley Tejada et al., 2020). Similarly, periods of touch deprivation have been associated with increased loneliness and a stronger desire for interpersonal physical contact (von Mohr et al., 2021). At the same time, other studies have found that individuals reporting greater loneliness express less liking for observed interpersonal touch (Saporta et al., 2022), highlighting the complex and potentially bidirectional relationship between loneliness and tactile experience.

A useful framework for interpreting these findings requires distinguishing among state, trait-like, and chronic loneliness. State loneliness refers to a transient response to a perceived discrepancy between desired and actual social connection and may temporarily increase motivation to seek proximity and restore social contact. Trait-like loneliness reflects relatively generalized individual differences in the tendency to perceive one’s social relationships as insufficient, whereas chronic loneliness entails the persistence of such experiences over time and may become accompanied by interpersonal distrust, negative social expectations, and heightened sensitivity to social threat (Qualter et al., 2015; Packheiser et al., 2025). The CLS used in the present study assesses generalized feelings of loneliness and social dissatisfaction in peer relationships rather than momentary loneliness elicited by a specific event (Asher et al., 1984; Maes et al., 2017). It can therefore be considered a trait-like measure. However, because it was administered only once and does not establish the duration or temporal stability of participants’ feelings, higher CLS scores should not be interpreted as evidence of chronic loneliness. Accordingly, the present findings indicate that adolescents reporting greater generalized peer-related loneliness showed different affective and cardiac responses to sensory stimulation, but they do not establish whether this pattern characterizes transiently or chronically lonely individuals.

This distinction may be relevant to the regulatory value of touch. Transient or non-entrenched feelings of social disconnection may heighten the motivation for reconnection, making bodily contact and interpersonal proximity particularly salient. By contrast, chronic loneliness may be accompanied by increasingly negative social expectations and interpersonal distrust, potentially reducing the perceived pleasantness or safety of social touch (Packheiser et al., 2025). Because the present study did not assess momentary loneliness, the duration of loneliness, or participants’ subjective appraisal of touch, it cannot distinguish between these possible mechanisms. Future longitudinal studies should combine repeated assessments of generalized loneliness with state measures administered before and after social exclusion, as well as ratings of touch pleasantness and comfort, to determine how the temporal course of loneliness shapes responsiveness to affective touch.

From a developmental perspective, early adolescence may represent a particularly important period in which these regulatory processes remain highly malleable. During this stage, social expectations, interpersonal trust, and emotion regulation are still undergoing substantial refinement. Interventions leveraging embodied forms of social interaction, such as affective touch, may therefore help support adaptive recovery from experiences of social exclusion before transient feelings of loneliness consolidate into more persistent patterns of social withdrawal and distrust.

### 4.1 Limitations of the study and future directions

Several methodological considerations should be acknowledged when interpreting the present findings. First, the tactile and auditory conditions differed along multiple dimensions beyond sensory modality alone. Affective touch involved direct bodily contact and conveyed interpersonal proximity and social significance. By contrast, the auditory stimulus provided a salient sensory event without physical contact or an inherently relational component. Consequently, the present design does not allow us to determine which of these characteristics or which combination of them, primarily accounted for the observed regulatory effects.

Importantly, the auditory condition was not intended to provide a perfectly matched sensory control. Rather, it was included to test whether the buffering effects previously observed following touch (Della Longa et al., 2026) could be explained by exposure to a salient sensory stimulus per se. The present findings argue against this interpretation. They instead suggest that a sensory experience combining bodily engagement with interpersonal meaning exerts regulatory effects that differ from those elicited by auditory stimulation alone.

Our previous work in adults (Della Longa et al., 2026) further supports this interpretation. Different forms of tactile stimulation varying in CT-optimal features and social contact, including hand stroking, brushing, and tapping, produced comparable reductions in subjective distress and autonomic arousal following social exclusion. By contrast, these effects were not observed in the absence of touch. Together, these findings suggest that the buffering effects of touch are unlikely to depend exclusively on specific tactile characteristics or on the pleasantness associated with a particular form of stimulation. Nevertheless, bodily contact, interpersonal proximity, and social meaning remain inherently intertwined during touch and cannot be fully disentangled within the present design. Future studies should therefore employ more tightly controlled comparison conditions. For example, they could manipulate the relational context while keeping tactile stimulation constant to clarify the respective contributions of bodily contact, sensory stimulation, and social.

Another related methodological consideration concerns the touch manipulation, which was delivered by the same trained female experimenter to all participants to minimize variability across experimental sessions. Nevertheless, touch delivered by an unfamiliar adult may be experienced differently depending on individual characteristics, including participants’ gender, interpersonal comfort, prior experiences with physical contact, and the meaning attributed to touch, particularly during adolescence. To minimize potential discomfort, participants were informed before the experiment that they would receive tactile stimulation, were shown how and where the touch would be delivered and were explicitly asked whether they were comfortable with this procedure. Furthermore, tactile stimulation was administered while participants were immersed in the virtual environment, preventing them from seeing the person delivering the touch. Although this procedure reduced the visual contextual cues typically associated with interpersonal touch, it could not eliminate the participants’ awareness that another person was physically present and providing the tactile stimulation. Importantly, this limitation also highlights a defining feature of affective touch: in natural social interactions, bodily contact and relational context are inherently intertwined. Thus, although the present findings demonstrate regulatory effects even when touch was delivered by an unfamiliar experimenter under highly controlled conditions, they do not allow us to determine how these effects would vary as a function of the relationship between the toucher and the recipient. Future studies should directly compare touch delivered by unfamiliar and familiar interaction partners, such as parents, close friends, or peers, to clarify how interpersonal context shapes the regulatory value of affective touch.

Additionally, the present study did not include a post-stimulation manipulation check assessing participants’ subjective experience of the tactile stimulation. Although participants were informed about the procedure, shown how and where the touch would be delivered, and asked beforehand whether they felt comfortable receiving it, we did not assess whether they subsequently experienced the touch as pleasant, soothing, comforting, socially supportive, awkward, or uncomfortable. Consequently, the observed affective and cardiac changes can be attributed to the tactile condition as administered, but the present data cannot establish whether these effects were driven by tactile stimulation itself, by its perceived pleasantness or supportive meaning, or by an interaction between sensory input and subjective appraisal. The absence of these measures also prevents us from determining whether individual differences in touch appraisal contributed to the associations observed between loneliness and responses to tactile stimulation. Future studies should therefore include trial-level or post-stimulation ratings of pleasantness, comfort, soothing value, and perceived social support to clarify how subjective appraisal contributes to the emotional and autonomic effects of touch following social exclusion.

Second, several methodological considerations relate to the social exclusion paradigm itself. We assessed emotional responses using broad dimensions of affective experience, namely valence and arousal, which capture core aspects of emotional regulation but may not fully reflect the multifaceted impact of ostracism. Social exclusion is known to affect higher-order psychological needs, such as belongingness, self-esteem, meaningful existence, and perceived control (e.g., as assessed using the Need-Threat Scale; Jamieson et al., 2010). Incorporating these measures would provide a more comprehensive characterization of the psychological consequences of exclusion. In addition, we adopted a single-session exclusion paradigm in which post-exclusion responses were compared with a pre-game baseline. This approach was chosen to maximize participants’ expectations of reciprocal interaction while minimizing habituation effects that may arise when repeatedly alternating between inclusion and exclusion blocks. Nevertheless, designs directly contrasting inclusion and exclusion conditions (e.g., von Mohr et al., 2017) may offer a more sensitive assessment of the affective and physiological consequences of ostracism and should be directly compared with the present approach in future studies.

The use of immersive virtual reality represents both a strength and a limitation of the present study. On the one hand, VR increases ecological validity by allowing participants to interact with avatars using embodied movements rather than simple button presses, thereby approximating natural social exchanges more closely than traditional Cyberball paradigms. On the other hand, virtual interactions may not always be experienced as fully equivalent to real interpersonal encounters. Likewise, the Cyberball paradigm does not unequivocally distinguish between intentional and unintentional exclusion, leaving room for participants to interpret the behavior of the other players in different ways (Jamieson et al., 2010; Rubo and Munsch, 2024). These factors may have attenuated the physiological impact of the exclusion manipulation. Consistent with this interpretation, participants reported a clear deterioration in their affective state following exclusion, whereas cardiac measures did not show a robust autonomic stress response. Rather than indicating that the paradigm failed to induce social distress, this dissociation may suggest that subjective and autonomic responses differ in their sensitivity to relatively mild forms of virtual exclusion. Future studies should therefore increase the social salience of the manipulation. For example they may use personalized avatars, familiar peers, or more explicit forms of rejection and complement cardiac measures with additional physiological markers, such as skin conductance, cortisol, or blood pressure to obtain a more comprehensive characterization of stress responses.

Another limitation concerns the feasibility-based sample size and the absence of a formal *a priori* power analysis. This is particularly relevant to the exploratory analyses examining the relationship between loneliness and the modulation of affective and autonomic responses. To our knowledge, the present study provides preliminary evidence suggesting that adolescents reporting higher levels of loneliness may show greater emotional and autonomic responsiveness to affective touch following social exclusion. However, these findings were based on condition-specific correlations conducted within relatively small subgroups. Moreover, no correction for multiple comparisons was applied. The observed associations were modest, and the study was not specifically powered to investigate individual differences or formally test loneliness as a moderator. Accordingly, these correlations should be regarded as preliminary and hypothesis-generating rather than confirmatory. Future studies should directly examine whether loneliness moderates the regulatory effects of affective touch using larger, adequately powered samples and preregistered inferential analyses.

Future investigations may also benefit from incorporating neural measures to better characterize the mechanisms underlying these regulatory effects. Electrophysiological indices such as the P3 component have been shown to distinguish social inclusion from exclusion and to reflect the allocation of attention to socially salient events (Weschke and Niedeggen, 2015; Jenkins and Obhi, 2020). Likewise, neuroimaging studies implicate regions involved in self–other representation and social connectedness, including the medial prefrontal cortex, patterns of activity which have been associated with perceived interpersonal closeness and loneliness (Courtney and Meyer, 2020). Combining neural measures with subjective and autonomic indices would provide a more comprehensive understanding of how affective touch promotes recovery following social exclusion, helping to clarify how bodily experience, social connectedness, and autonomic regulation are integrated at both central and peripheral levels.

Finally, the present study focused on typically developing young adolescents during a developmental period characterized by heightened sensitivity to peer evaluation, substantial neural plasticity, and ongoing maturation of social regulatory processes. Although this focus was theoretically motivated, the findings may not generalize to populations with atypical neurodevelopment (e.g., individuals with autism spectrum disorder) or to adolescents experiencing persistent social withdrawal (e.g., hikikomori). At the same time, these developmental characteristics suggest that adolescence may represent a particularly promising window for interventions targeting embodied forms of social regulation. If replicated and extended, the present findings may ultimately inform preventive approaches aimed at strengthening resilience to social exclusion and reducing the risk that transient experiences of loneliness will evolve into more persistent patterns of social disconnection.

## 5 Conclusion

The present study provides preliminary evidence that affective touch may promote emotional recovery and autonomic downregulation following virtual social exclusion, as well as autonomic downregulation during early adolescence. Compared with a non-social auditory stimulus, affective touch was associated with reduced sympathetic activation and greater parasympathetic engagement, and a more complete restoration of affective state. This pattern is consistent with a shift toward autonomic downregulation and suggests that not all sensory experiences are equally effective in facilitating recovery from social threats.

Importantly, exploratory analyses indicated that these regulatory effects were particularly pronounced in adolescents reporting higher levels of loneliness, raising the possibility that affective touch may be especially beneficial when the need for social connection is heightened. More broadly, the present findings support the view that the regulatory value of affective touch extends beyond its sensory properties. By simultaneously engaging the body and conveying interpersonal closeness, affective touch may represent a unique form of embodied social experience capable of restoring emotional and autonomic balance following social exclusion.

From a developmental perspective, these findings highlight the importance of considering the body not merely as the recipient of sensory stimulation, but as a primary medium through which social connection is experienced and restored during adolescence. A better understanding of how the bodily and relational aspects of touch interact to promote resilience may ultimately inform preventive interventions aimed at reducing the long-term consequences of loneliness and social exclusion during this sensitive developmental period.

## Data Availability

The name of the repository and accession number(s) can be found in Open Science Framework (OSF) at https://osf.io/uqs4p/overview. Any additional information required to reanalyze the data reported in this paper is available from the lead contact upon request.
