# Supplementary material for: Touch promotes emotional and autonomic recovery following virtual social exclusion in early adolescents
Source: Front Psychol. 2026 Oct 23;17:1981624. doi: 10.3389/fpsyg.2026.1981624 (PMC13739663; doi:10.3389/fpsyg.2026.1981624)
Supplement: Supplementary file 1 [file Supplementary_file_1.DOCX]

Supplementary Material

1. **Sample description and missing data**

| Group | Physiological dataset (n) | Missing physiological data (n) | Subjective measures dataset (n) | Missing Subjective data (n) | Loneliness dataset (n) | Missing  Loneliness data (n) |
| --- | --- | --- | --- | --- | --- | --- |
| Touch | 28 | 8 | 35 | 1 | 30 | 6 |
| Sound | 29 | 4 | 33 | 0 | 33 | 0 |

**Supplementary Table 1***.* The table reports the number of participants with available data and missing data for each outcome measure.

2. **Preliminary analysis on baseline indices**

We controlled for potential confounding individual differences in the two groups of participants by running preliminary analysis on baseline levels of subjective measures (valence and arousal) and cardiac indices (heart rate - HR, heart rate variability - RMSSD index, sympathetic activation - SNS, parasympathetic activation - PNS). Table S2 presents descriptive statistics for each group and the results of independent-samples *t*-tests assessing between-group differences in baseline subjective and physiological measures. No significant differences emerged between the two groups for any of the baseline measures, suggesting that the groups did not systematically differ in subjective or physiological indices prior to the exclusion manipulation.

|  | **Group** | **Mean (SD)** | **T-test** |
| --- | --- | --- | --- |
| **Valence** | Touch | 1.53 (0.68) | t= 0.197, p= 0.844 |
|  | Sound | 1.56 (0.63) |  |
| **Arousal** | Touch | -1.16 (0.87) | t= -0.249, p= 0.804 |
|  | Sound | -1.10 (1.02) |  |
| **HR** | Touch | 87.90 (11.77) | t= -0.710, p= 0.481 |
|  | Sound | 85.67 (11.70) |  |
| **RMSSD** | Touch | 38.11 (22.20) | t= 0.622, p= 0.536 |
|  | Sound | 42.54 (30.84) |  |
| **SNS** | Touch | 2.40 (1.74) | t= -0.415, p=0.680 |
|  | Sound | 2.20 (1.80) |  |
| **PNS** | Touch | -1.14 (1.02) | t= 0.869, p=0.389 |
|  | Sound | -0.87 (1.28) |  |

**Supplementary Table 2.** Descriptive statistics and analysis of baseline subjective (valence and arousal) and physiological measures (HR, RMSSD, SNS, PNS).

3. **Preliminary analysis on loneliness scores**

To control for potential differences in loneliness between the two groups of participants, we conducted a preliminary analysis of Children’s Loneliness Scale (CLS) scores. Table S3 presents descriptive statistics for each group and the results of an independent-samples *t*-test assessing between-group differences. No significant difference in loneliness scores emerged between the two groups, indicating comparable levels of loneliness across participants in the two experimental conditions.

|  | **N** | **Mean (SD)** | **Range** | **T-test** |
| --- | --- | --- | --- | --- |
| **Touch** | 33 | 29.8 (9.14) | 18-55 | t= 0.33, p= 0.741 |
|  |  |  |  |  |
| **Sound** | 29 | 30.6 (9.30) | 18-56 |  |
|  |  |  |  |  |
| **All** | 62 | 30.2 (9.16) | 18-56 |  |
|  |  |  |  |  |

**Supplementary Table 3.** Descriptive statistics and analysis of loneliness scores.

4. **Model comparison analyses on Valence**

| Tested  models | Variables | AIC | wAIC | cond R^2^ | marg R^2^ |
| --- | --- | --- | --- | --- | --- |
| Model 0 | Random effect of Participants | 277.04 | 0.000 | 0.189 | 0.000 |
| Model 1 | + Phase | 267.75 | 0.041 | 0.321 | 0.070 |
| Model 2 | + Group | 269.31 | 0.019 | 0.328 | 0.073 |
| Model 3 | + Phase × Group | 265.52 | 0.126 | 0.382 | 0.103 |
| Model 4 | + Gender | 263.91 | 0.280 | 0.387 | 0.136 |
| Model 5 | + Age | 262.63 | 0.533 | 0.392 | 0.163 |

**Supplementary Table 4.** Comparison between models predicting valence modulation. Note that smaller values of AIC indicate better fitting models.

**ANOVA on model 5**

             	Chisq	Df	Pr(>Chisq)

(Intercept)	5.5396 	1   	0.01859 *

Phase      	0.7342 	1   	0.39153

Group     	0.5298 	1  	0.46670

Gender     	1.8250 	1  	0.17672

Age        	3.1516 	1  	0.07585 .

Phase × Group	5.8734 	1   	0.01537 *

**Supplementary Figure 1***.* Plot showing the modulation of valence across the phases of the experiment and between the two groups.

5. **Model comparison analyses on Arousal**

| Tested  models | Variables | AIC | wAIC | cond R^2^ | marg R^2^ |
| --- | --- | --- | --- | --- | --- |
| Model 0 | Random effect of Participants | 329.77 | 0.001 | 0.421 | 0.000 |
| Model 1 | + Phase | 331.52 | 0.000 | 0.417 | 0.001 |
| Model 2 | + Group | 333.08 | 0.000 | 0.423 | 0.006 |
| Model 3 | + Phase × Group | 315.74 | 0.655 | 0.570 | 0.081 |
| Model 4 | + Gender | 317.72 | 0.244 | 0.575 | 0.081 |
| Model 5 | + Age | 319.48 | 0.101 | 0.580 | 0.082 |

**Supplementary Table 5.** Comparison between models predicting arousal modulation. Note that smaller values of AIC indicate better fitting models.

**ANOVA on model 3**

             	Chisq	Df	Pr(>Chisq)

(Intercept) 	1.4139 	1  	0.234402

Phase       	8.2254 	1  	0.004131 **

Group       	2.8735 	1 	0.090048 .

Phase × Group	21.9200 	1 	2.843e-06 ***

**Supplementary Figure 2.** Plot showing the modulation of arousal across the phases of the experiment and between the two groups.

6. **Model comparison analyses on HR**

| Tested  models | Variables | AIC | wAIC | cond R^2^ | marg R^2^ |
| --- | --- | --- | --- | --- | --- |
| Model 0 | Random effect of Participants | 624.69 | 0.000 | 0.443 | 0.000 |
| Model 1 | + Phase | 611.97 | 0.000 | 0.566 | 0.065 |
| Model 2 | + Group | 607.90 | 0.000 | 0.571 | 0.136 |
| Model 3 | + Phase × Group | 591.26 | 0.470 | 0.687 | 0.196 |
| Model 4 | + Gender | 591.82 | 0.354 | 0.691 | 0.210 |
| Model 5 | + Age | 593.22 | 0.176 | 0.691 | 0.194 |

**Supplementary Table 6.** Comparison between models predicting HR modulation. Note that smaller values of AIC indicate better fitting models.

**ANOVA on model 3**

             	Chisq	Df	Pr(>Chisq)

(Intercept) 	8.2433 	1   	0.00409 **

Phase       	0.1125 	1   	0.73734

Group       	0.0380 	1   	0.84547

Phase × Group	21.3337 	1 	3.859e-06 ***

**Supplementary Figure 3***.* Plot showing the modulation of HR across the phases of the experiment and between the two groups.

7. **Model comparison analyses on RMSSD**

| Tested  models | Variables | AIC | wAIC | cond R^2^ | marg R^2^ |
| --- | --- | --- | --- | --- | --- |
| Model 0 | Random effect of Participants | 624.69 | 0.004 | 0.291 | 0.000 |
| Model 1 | + Phase | 611.97 | 0.390 | 0.414 | 0.065 |
| Model 2 | + Group | 607.90 | 0.242 | 0.421 | 0.076 |
| Model 3 | + Phase × Group | 591.26 | 0.175 | 0.427 | 0.083 |
| Model 4 | + Gender | 593.22 | 0.067 | 0.434 | 0.082 |
| Model 5 | + Age | 593.66 | 0.123 | 0.439 | 0.115 |

**Supplementary Table 7***.* Comparison between models predicting RMSSD modulation. Note that smaller values of AIC indicate better fitting models.

**ANOVA on model 1**

             	Chisq	Df	Pr(>Chisq)

(Intercept) 	0.0763 	1 	0.7823694

Phase      	12.3831 	1 	0.0004332 ***

**Supplementary Figure 4***.* Plot showing the modulation of RMSSD across the phases of the experiment and between the two groups.

8. **Model comparison analyses on SNS**

| Tested  models | Variables | AIC | wAIC | cond R^2^ | marg R^2^ |
| --- | --- | --- | --- | --- | --- |
| Model 0 | Random effect of Participants | 235.73 | 0.000 | 0.670 | 0.000 |
| Model 1 | + Phase | 212.87 | 0.004 | 0.785 | 0.060 |
| Model 2 | + Group | 206.61 | 0.085 | 0.787 | 0.171 |
| Model 3 | + Phase × Group | 203.83 | 0.342 | 0.802 | 0.179 |
| Model 4 | + Gender | 203.47 | 0.408 | 0.804 | 0.205 |
| Model 5 | + Age | 205.33 | 0.161 | 0.804 | 0.183 |

**Supplementary Table 8***.* Comparison between models predicting SNS modulation. Note that smaller values of AIC indicate better fitting models.

**ANOVA on model 4**

             	Chisq	Df	Pr(>Chisq)

(Intercept) 	1.9355 	1  	0.164158

Phase      	7.2938 	1  	0.006919 **

Group      	5.3739 	1  	0.020440 *

Age        	2.2771 	1  	0.131293

Phase × Group	4.8103 	1  	0.028290 *

**Supplementary Figure 5***.* Plot showing the modulation of SNS across the phases of the experiment and between the two groups.

9. **Model comparison analyses on PNS**

| Tested  models | Variables | AIC | wAIC | cond R^2^ | marg R^2^ |
| --- | --- | --- | --- | --- | --- |
| Model 0 | Random effect of Participants | 162.32 | 0.000 | 0.261 | 0.000 |
| Model 1 | + Phase | 149.48 | 0.023 | 0.426 | 0.086 |
| Model 2 | + Group | 149.19 | 0.026 | 0.432 | 0.110 |
| Model 3 | + Phase × Group | 145.76 | 0.145 | 0.478 | 0.135 |
| Model 4 | + Gender | 142.96 | 0.589 | 0.482 | 0.182 |
| Model 5 | + Age | 144.95 | 0.217 | 0.488 | 0.180 |

**Supplementary Table 9***.* Comparison between models predicting PNS modulation. Note that smaller values of AIC indicate better fitting models.

**ANOVA on model 4**

             	Chisq	Df	Pr(>Chisq)

(Intercept) 	4.2882 	1  	0.03838 *

Phase      	2.2891 	1   	0.13029

Group      	0.2017 	1   	0.65332

Age        	4.7482 	1   	0.02933 *

Phase × Group	5.4879 	1   	0.01915 *

 **Supplementary Figure 6***.* Plot showing the modulation of PNS across the phases of the experiment and between the two groups (A) and the effect of Age (B).
